# Population coding in mouse visual cortex: response reliability and dissociability of stimulus tuning and noise correlation

**DOI:** 10.3389/fncom.2014.00058

**Published:** 2014-06-02

**Authors:** Jorrit S. Montijn, Martin Vinck, Cyriel M. A. Pennartz

**Affiliations:** ^1^Cognitive and Systems Neuroscience, Faculty of Science, Center for Neuroscience, Swammerdam Institute for Life Sciences, University of Amsterdam Amsterdam, Netherlands; ^2^Research Priority Program Brain and Cognition, University of Amsterdam Amsterdam, Netherlands

**Keywords:** population coding, calcium imaging, spatial organization, mouse, visual cortex, orientation tuning, noise correlations, signal correlations

## Abstract

The primary visual cortex is an excellent model system for investigating how neuronal populations encode information, because of well-documented relationships between stimulus characteristics and neuronal activation patterns. We used two-photon calcium imaging data to relate the performance of different methods for studying population coding (population vectors, template matching, and Bayesian decoding algorithms) to their underlying assumptions. We show that the variability of neuronal responses may hamper the decoding of population activity, and that a normalization to correct for this variability may be of critical importance for correct decoding of population activity. Second, by comparing noise correlations and stimulus tuning we find that these properties have dissociated anatomical correlates, even though noise correlations have been previously hypothesized to reflect common synaptic input. We hypothesize that noise correlations arise from large non-specific increases in spiking activity acting on many weak synapses simultaneously, while neuronal stimulus response properties are dependent on more reliable connections. Finally, this paper provides practical guidelines for further research on population coding and shows that population coding cannot be approximated by a simple summation of inputs, but is heavily influenced by factors such as input reliability and noise correlation structure.

## Introduction

Stimuli in an animal's environment are encoded by the pattern of action potential firing of a neuron (Hubel and Wiesel, 1974). It remains unclear however how precisely information about the outside world is encoded and processed in the brain on a network level. Using information coded by single neurons is straightforward, but when the activity of multiple neurons is used to code information, higher-order statistics such as temporal dependencies can strongly influence neural codes (Butts et al., 2007; Ainsworth et al., 2012).

To study these general principles of population coding, it is necessary to choose a suitable model system. For many years, the primary visual cortex has been serving as such a model (Tong, 2003). Many properties of V1 appear to be preserved across mammalian species, including neuronal tuning properties, but the spatial organization of these neurons differs. For example, in primates orientation tuned neurons are organized in columns (Hubel and Wiesel, 1974), while in mouse V1 neurons that are tuned to different orientations are organized in an intermingled “salt-and-pepper” way (Hübener, 2003). Despite the apparent absence of orientation columns in mouse V1, pyramidal neurons still exhibit selectivity to many visual stimulus properties, such as retinal location, orientation or motion direction, contrast, spatial and temporal frequency, and speed (Hübener, 2003; Niell and Stryker, 2008; Andermann et al., 2011). These factors, in combination with the relative ease to work with mice and the many options for genetic modifications, make them an excellent animal model for studying population coding of visual information.

One of the biggest challenges of population coding is understanding how higher-order neuronal populations can read out their input from primary sensory populations, especially considering the relatively high noise of neural systems (Faisal et al., 2008), and the complex spatiotemporal structures this noise can assume (correlated trial-to-trial variability between neurons that occurs regardless of any similarity in the mean response of these neurons to stimuli, i.e., noise correlations) (Cohen and Kohn, 2011). Even when disregarding noise, this reading-out—or decoding—is non-trivial, since neurons are often responsive to a plethora of stimulus properties, while higher-level populations need to extract specific information from this entangled stimulus representation (Pagan et al., 2013). In addition to the already large number of stimulus dimensions that influence responses of neurons in V1, their firing patterns can be modulated by non-stimulus related processes such as attention (Motter, 1993), wakefulness (Greenberg et al., 2008), reward-related activity (Goltstein et al., 2013), or even higher-order properties of the visual scene, such as whether an object belongs to the foreground or background of a scene (Lamme, 1995).

One way to study population coding is with the use of computational algorithms that decode information from the population activity (e.g., Zhang et al., 1998; Pillow et al., 2008; Quiroga and Panzeri, 2009; Graf et al., 2011). Comparing a decoder's performance in different situations can then provide insight into how much information is present in population spiking activity. Moreover, comparing the performance of different decoders may provide insight into how population coding works in the brain and into which variables are important. Using decoding algorithms has been particularly fruitful in the investigation of the effects that specific spatiotemporal structures of noise (noise correlations) have on population coding (Pillow et al., 2008; Graf et al., 2011), although it remains unclear whether they are beneficial (e.g., Averbeck et al., 2006; Ecker et al., 2011; da Silveira and Berry, 2013) or detrimental (e.g., Mitchell et al., 2009). Moreover, it is still unknown whether the same anatomical substrates (i.e., synaptic connections) underlie both noise correlations and stimulus tuning. It has been hypothesized for some years that noise correlations are dependent on horizontal cortico-cortical connections (Ts'o et al., 1986; Smith et al., 2012), and it has more recently been shown that L2/3 orientation tuned neurons have a higher intra-laminar connection probability with similarly tuned neurons (Ko et al., 2011). This would make it plausible that these two processes indeed share similar anatomical substrates, but this hypothesis has—to our knowledge—not yet been tested.

A number of decoding algorithms has been applied to neural data recorded from several brain regions, but to our knowledge the performance of these algorithms has not been compared in mouse primary visual cortex (V1). Especially the influence of single neurons on the decoding performance of ensembles has been studied little, even though it has been known for several years that neuronal populations in V1 show sparse coding, where single attributes in a visual scene are represented by only a very small number of neurons (Vinje and Gallant, 2000). We will therefore compare the performance of a classical population vector, template matching, variability-normalizing template matching, and Bayesian maximum-likelihood algorithm as a function of neuronal population size. We also relate their performance to their mathematical assumptions to show which variables determine the applicability of the different decoders. We performed these analyses using population data obtained by *in vivo* two-photon calcium imaging of ensemble activity in V1, which directly reflects spiking activity (Kerr et al., 2005; Greenberg et al., 2008). Furthermore, we investigate the dependence of decoding accuracy on orientation selectivity, signal correlations and noise correlations, and show the relationships between these neuronal properties. Methodological descriptions of several analysis methods are provided so they can be used as guidelines by other researchers for future investigation of population coding. The results from these analyses reveal the caveats and critical factors of applying population vector and template matching algorithms to population activity of V1 neurons, including the pivotal importance of response reliability (i.e., little variation over trials in response magnitude). Secondly, our results show that there exists an anatomical intersomatic distance dependence of noise correlations, but not of the difference in preferred visual stimulus direction between pairs of cells. These results lead us to formulate two hypotheses for which we also present testable predictions. First, we argue that a Hebbian plasticity mechanism might lead to a normalization of neuronal responses based on their variability. Secondly, we hypothesize that noise correlations are due to global non-specific synaptic barrages in local circuits, while stimulus response properties are dependent on stronger or more reliable connections.

## Methods

### Animal preparation

All experimental procedures were conducted with approval of the animal ethics committee of the University of Amsterdam and are largely similar to those described in more detail in Goltstein et al. (2013). In short, 6 adult male C57BL/6 mice (Harlan) were implanted with a titanium head bar prior to the imaging experiment and were allowed to recover for a minimum of 3 days. On the day of the two-photon calcium imaging experiment, buprenorphine (0.05 mg/kg) was injected subcutaneously 30–60 min before induction of anesthesia with isoflurane (3.0% in 100% oxygen). Intrinsic signal imaging (ISI) was performed to localize the primary visual cortex (V1) while the animal was anesthetized lightly (0.8% isoflurane). We subsequently performed a small (2 mm) craniotomy above the retinotopic area responding to visual stimulation with drifting gratings. After the craniotomy, the dura was kept wet with an artificial cerebrospinal fluid (ACSF: NaCl 125 mM, KCl 5.0 mM, MgSO_4_ * 7 H_2_O 2.0 mM, NaH_2_PO_4_ 2.0 mM, CaCl_2_ * 2 H_2_O 2.5 mM, glucose 10 mM) buffered with HEPES (10 mM, adjusted to pH 7.4) (Svoboda et al., 1999). After removal of the skull, multi-cell bolus loading with Oregon Green BAPTA-1 AM (OGB) and Sulforhodamine 101 (SR101) was performed 230–270 microns below the dura as previously described (Stosiek et al., 2003; Goltstein et al., 2013). After injection of the dyes, the exposed dura was covered with agarose (1.5% in ACSF) and sealed with a circular cover glass that was fixed to the skull using cyanoacrylate glue.

### Apparatus and stimulus presentations

**Figure 1:**
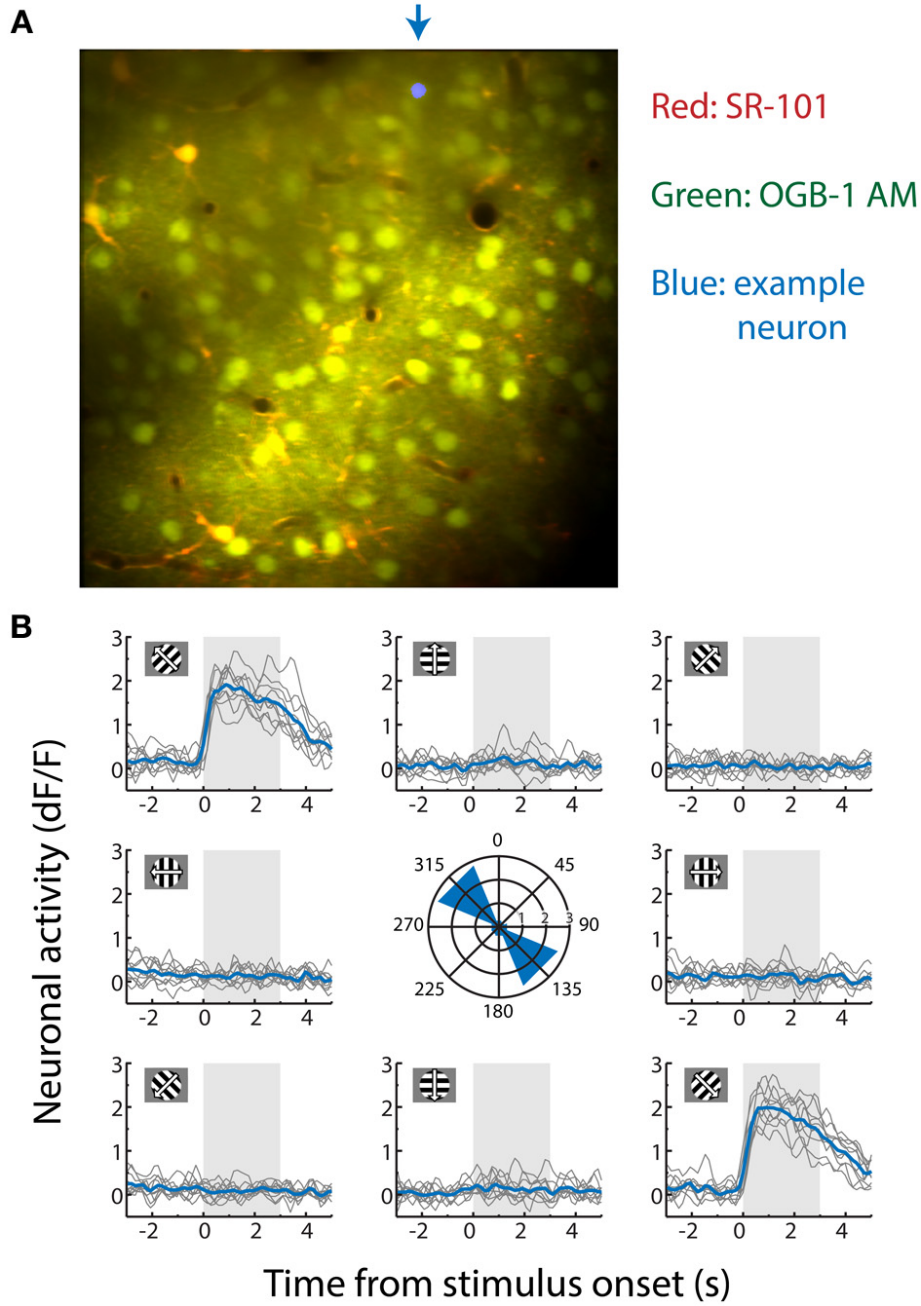
**(A)** Contrast-enhanced average of all x-y movement-corrected frames recorded from an anesthetized mouse during a two-photon calcium imaging experiment used for decoding. The data were obtained by recording fluorescence levels in neurons stained with Oregon Green BAPTA-1 AM (OGB-1 AM). Dual dye-loading with Sulforhodamine-101 (SR-101) was performed to differentiate astrocytes (yellow/orange) from neuronal cell bodies (green). The location marked in blue in the upper part of the example recording is the cell body of the neuron whose stimulus-selective responses are shown in **(B)**. **(B)** Activity trace of an example neuron measured by the *dF/F*_0_ (fraction change in fluorescence level relative to a 30 s baseline, which correlates approximately linearly with spiking activity). This neuron responds strongly to gratings moving to the upper left (315°) and lower right (135°), but not to any other moving direction. It is therefore strongly orientation-tuned, but weakly direction-tuned. Each gray line depicts a single trial (−3 to +5 s of stimulus onset) and the mean response per stimulus direction over all ten repetitions is shown in blue. The area shaded in gray shows stimulus presentation time. Depicted in the center graph is the maximum value of the mean response of the neuron per stimulus direction in *dF/F*_0_.

Dual-channel two-photon imaging recordings (filtered at 500–550 nm for OGB and 565–605 nm for SR101; see Figure 1A) with a 512 × 512 pixel frame size were performed at a sampling frequency of 25.4 Hz. We used an *in vivo* two-photon laser scanning microscopy setup (modified Leica SP5 confocal system) with a Spectra-Physics Mai-Tai HP laser set at a wavelength of 810 nm to simultaneously excite OGB and SR101 molecules, as previously described (Goltstein et al., 2013). Mice (*n* = 6) were kept lightly anesthetized (0.8% isoflurane) during the entirety of the two-photon calcium imaging recordings (*n* = 6, 1 data set/animal), while they were presented with 10 repetitions of 8 different directions of square-wave drifting gratings (*n* = 80 trials/recording). Visual stimulation duration was 3 s and was alternated by a 5 s blank inter-trial interval during which an isoluminant gray screen was presented. Visual drifting gratings (diameter 60 retinal degrees, spatial frequency 0.05 cycles/degree, temporal frequency 1 Hz) were presented within a circular cosine-ramped window to avoid edge effects at the border of the circular window. All visual stimulation was performed on a 15 inch TFT screen with a refresh rate of 60 Hz positioned at 16 cm from the mouse's eye, which was controlled by MATLAB using the PsychToolbox extension (Brainard, 1997; Pelli, 1997). A field-programmable gate array (FPGA, OpalKelly XEM3001) was connected to the microscope setup and interfaced with the stimulus computer to synchronize the timing of the visual stimulation with the microscope frame acquisition.

### Data preprocessing

After a recording was completed small x-y drifts were corrected with an image registration algorithm (Guizar-Sicairos et al., 2008) and the recording was manually checked for movement artifacts along the z-axis. If z-drifts occurred during a recording, it was rejected and no further analyses were performed. Regions of interest (neurons, astrocytes, and blood vessels) were determined semi-automatically using custom-made MATLAB software and subsequently *dF/F*_0_ values for all neurons were calculated as previously described (Goltstein et al., 2013). In short, for each image frame *i* a single *dF/F*_0_ value was obtained for each neuron by calculating the baseline fluorescence (*F*_0*i*_), taken as the mean of the lowest 50% during a 30-s window preceding image frame *i*. *dF* is defined as the difference between the fluorescence for that neuron in the given frame and the sliding baseline fluorescence (*d*F_i_ = F_i_−F_0*i*_) (Goltstein et al., 2013). The mean number of simultaneously recorded neurons/session was 118 (range: 95–144).

### Neuronal responses to visual stimuli

A neuron's response to a certain stimulus was defined as the mean *dF/F*_0_ value of all frames recorded during that single trial's stimulus presentation (see Figure 1B for an example neuron). Stimulus presentation lasted 3.0 s (77 frames) and a single stimulus presentation yields a single response value *R*, equal to the mean *dF/F*_0_ over all 77 stimulus frames. To obtain a measure of a neuron's orientation selectivity, we calculated the orientation selectivity index (OSI) as (1-circular variance), as previously described by Ringach et al. (2002). To further parameterize neuronal orientation tuning, we also calculated each neuron's preferred direction by fitting a double von Mises distribution to the neuron's responses, where the peaks of both von Mises functions are opposite to each other (separated by 180°):

(1) $$f\text{​}\left(x|\theta ,\kappa_{1},\kappa_{2},\mu_{0}\right)=\frac{e^{\;\kappa_{1}\cos\text{​}(x\;-\;\theta )}}{2\pi I_{0}(\kappa_{1})}+\frac{e^{\;\kappa_{2}\cos\text{​}(x\;+\;\pi \;-\;\theta )}}{2\pi I_{0}(\kappa_{2})}+\mu_{0}$$

Here, *I*_0_(κ) is the modified Bessel function of order 0 and *x* represents the stimulus angle. As can be seen in the equation, we defined the free parameters as θ (preferred direction), κ_1_ (concentration parameter at θ), κ_2_ (concentration parameter at θ +π) and μ_0_ (baseline response). A neuron's preferred direction was defined as the angle with the highest concentration parameter (which could be either κ_1_ or κ_2_).

### Signal correlations

In many cases neuronal responses in V1 to drifting gratings can be fairly accurately approximated by circular von Mises distributions. However, in order to more fully capture similarities and differences between the responses that neurons show to drifting gratings of different directions, we calculated signal correlations between all neuronal pairs in each recording. We defined each direction as a separate stimulus type and calculated a neuron's mean response vector ***R***, where the elements of ***R*** are the neuron's mean responses to each direction θ (*R*_θ_):

(2) $$\bar{R}=[{\bar{R}}_{0},{\bar{R}}_{45}\ldots \;{\bar{R}}_{315}]$$

We then calculated the pairwise signal correlation as the Pearson correlation between two neurons' (*i*,*j*) response vectors:

(3) $$\rho_{i,j}^{\text{signal}}=\text{corr}({\bar{R}}_{i},{\bar{R}}_{j})$$

Since the decoding algorithms used to extract information from the population response depend on the difference in response between neurons for different stimulus directions, high signal correlations between two neurons indicate that there is a large redundancy in information that these neurons provide to the decoder.

### Noise correlations

Complementary to signal correlations, which provide an index of the similarity between pairs of neurons in their mean response to the different stimulus types, are noise correlations, which give an indication of the similarity in trial-by-trial response variability between neurons. We first calculated a response vector for each stimulus type θ, where each element in the vector is the neuron's response to a single presentation *i* of that stimulus direction:

(4) $$R_{\theta }=[R_{\theta_{i}}\ldots \;R_{\theta_{n}}],$$

where *n* is ten, since we have ten repetitions per direction. Because we aim to compare a single noise correlation value per neuronal pair, we took the mean noise correlation over all eight stimulus directions θ = 0°–315° (with steps of 45°):

(5) $$\rho_{i,j}^{\text{noise}}=\frac{\sum_{\theta \;=\;0}^{315}\text{corr}(R_{i,\theta },R_{j,\theta })}{8}$$

The noise correlation is therefore an index of the mean shared trial-by-trial variability over all stimulus directions.

### Decoding sample bootstrapping

All decoding algorithms (described in detail in the following paragraphs) were tested on their ability to recover the presented stimulus direction from the neuronal population activity during single trials. To quantify their dependence on the number of neurons included, we took random subsamples of neurons (100 iterations) from each data set (ranging from 95 to 144 neurons simultaneously recorded neurons) for all tested sample sizes (1–90 neurons, each neuron was included only once). For each random sample we trained the decoder on the activity of the randomly included neurons for all 80 trials (8 directions, 10 repetitions) and subsequently decoded the presented direction of each trial from the population activity of only these neurons. Since the smallest data set consisted of 95 neurons, we ran our resampling procedures up to a maximum population size of 90 neurons. We performed these resamplings independently for each decoding algorithm.

### Population vector decoding

To decode the presented stimulus direction during a given trial, we used the population vector method (Georgopoulos et al., 1986) as follows. For each trial, the activity of each neuron was represented as a vector, where the angle (θ) represents the neuron's preferred direction and the magnitude (ρ) corresponds to its activity level (*dF/F*_0_). A resultant population vector can then be calculated by taking the circular mean over all neurons. When the resultant angle was within 22.5 degrees of the actual stimulus direction, it was counted as a “correct” decoding, in all other cases it was marked as “incorrect.” After decoding all trials, the percentage correct decoded was then calculated over all trials, so it could be plotted as in Figure 4.

### Template matching algorithm

One step up in complexity from the population vector is the template matching algorithm (Lehky and Sejnowski, 1990; Zhang et al., 1998; van Duuren et al., 2008, 2009). While in the population vector method each neuron is represented only by a preferred direction and its activity level, template matching compares the population activity to response templates of different stimulus types. These templates are generated by taking the mean activity across trials during the presentation of each stimulus direction (θ) for each neuron, resulting in a “template population activity.” The similarity of this template (**R^θ^**) to the actual population activity (**R**^stim^) is given by

(6) $$\begin{array}{c} \Theta_{\theta }=\frac{\sum_{\text{i}=1}^{N}{\text{R}}_{i}^{\text{stim}}\cdot {\text{R}}_{i}^{\theta }}{\left\|{\text{R}}^{\theta }\|\cdot \|{\text{R}}^{\text{stim}}\prime \right\|}, \end{array}$$

where *i* indexes the *N* elements (neurons) of **R**, and · indicates multiplication. The similarity index Θ is calculated for all eight directions and the decoded output is determined by taking the direction with the highest similarity to the population activity.

### The non-scale sensitive fano factor (rFano)

The Fano factor provides an indication of the variability of neuronal responses. Two-photon calcium imaging yields neuronal responses that are given as *dF/F*_0_, where the relationship between the actual spiking rate and the *dF/F*_0_ is approximately linear per neuron, but varies over neurons (Kerr et al., 2005; Greenberg et al., 2008). Compared to firing rates, neuronal *dF/F*_0_ responses are therefore scaled by an arbitrary constant *c* by which both the mean (μ) and standard deviation (σ) of its responses over the entire recording are multiplied. The scaling sensitivity is described by the following equation:

(7) $$\text{Fano}=\frac{\sigma^{2}\cdot c^{2}}{\mu \cdot \text{c}}$$

Therefore, Fano factors calculated from calcium imaging data are only accurate when *c* = 1, as scaling (of the firing rate) has a disproportionally larger effect on the variance than the mean. For this reason, raw Fano Factors from calcium imaging cannot be simply translated to the Fano Factors of the raw firing rates. To correctly calculate the variability of calcium transient data, the variance and mean activity level per neuron must be normalized, thereby removing the constant *c* from the equation. While it is not possible to compute the raw Fano factors of the unknown firing rates, it is possible, however, to compute a Fano factor based on relative changes in mean and variance across stimulus dimensions. We therefore define the ratio Fano factor (r_Fano_) as follows:

(8) $$r_{\text{Fano}}=\frac{\sigma_{p}^{2}/\sigma_{np}^{2}}{\mu_{p}/\mu_{np}}$$

where μ_*p*_ and σ^2^_*p*_ are the mean response and variance over trials to the neuron's preferred (P°) and opposite (P° + 180°) direction, and μ_np_ and σ^2^_np_ are the mean response and variance to the other six moving directions. This way, the mean and variance are described as fractional increases in activity and variability from non-preferred stimulus epochs to preferred stimulus epochs. Because c^2^ appears in both variance terms and c appears in both mean response terms, the r_Fano_ factor is no longer scale sensitive.

### Normalized template matching

In an attempt to improve the performance of the template matching algorithm, we performed a similar decoding operation, but now on the z-scored rather than the raw *dF/F*_0_ activity values. The new equation becomes

(9) $$\Theta_{\theta }=\frac{\sum_{\text{i }=\;1}^{N}{\text{Z}}_{i}^{\text{stim}}\cdot {\text{Z}}_{i}^{\theta }}{\left\|Z^{\theta }\right\|\cdot \left\|Z^{\text{stim},}\right\|},$$

where *Z* is an index that describes a neuron's *dF/F*_0_ response (*R*) for any time point (*i*) as the number of standard deviations (σ) from the mean *dF/F*_0_ calculated over the entire recording (*R*_all_):

(10) $$Z_{i}=\frac{\left(R_{i}-{\bar{R}}_{\text{all}}\right)}{\sigma_{R_{\text{all}}}}$$

Therefore a neuron that shows a high mean activation level (*R*_all_), but an even higher variability σ_*R*_all__ (high Fano factor) will contribute less to the template similarity than a neuron with a low activation level and an even lower variability (low Fano factor).

### Bayesian maximum-likelihood (ML)

The final algorithm we used was a Bayesian decoder. Bayes' rule is given by

(11) $$P\text{​}\left(\theta |A_{\text{pop}}\right)=\frac{P\text{​}\left(A_{\text{pop}}|\theta \right)P\text{​}\left(\theta \right)}{P\text{​}\left(A_{\text{pop}}\right)},$$

where P(θ), the prior, is the prior probability of having direction θ; P(θ | *A*_pop_), the posterior, is the probability of this trial's population activity being caused by direction θ; P(*A*_pop_ | θ), the likelihood, represents the probability that stimulus direction θ will result in pattern *A*_pop_; and P(*A*_pop_) is the probability of activation pattern A_pop_, or model evidence—a normalization term. It has been shown before that there is little difference in performance between uniform and natural priors—at least for retinal ganglion cells (Jacobs et al., 2009)—so for the following results we assumed a uniform prior [P(θ) is equal for all directions] to simplify computational procedures. Note that P(*A*_pop_) is identical for all stimulus directions, since the term θ is not present. Therefore, for a given direction θ, the posterior probability is proportional to the likelihood.

**Figure 2:**
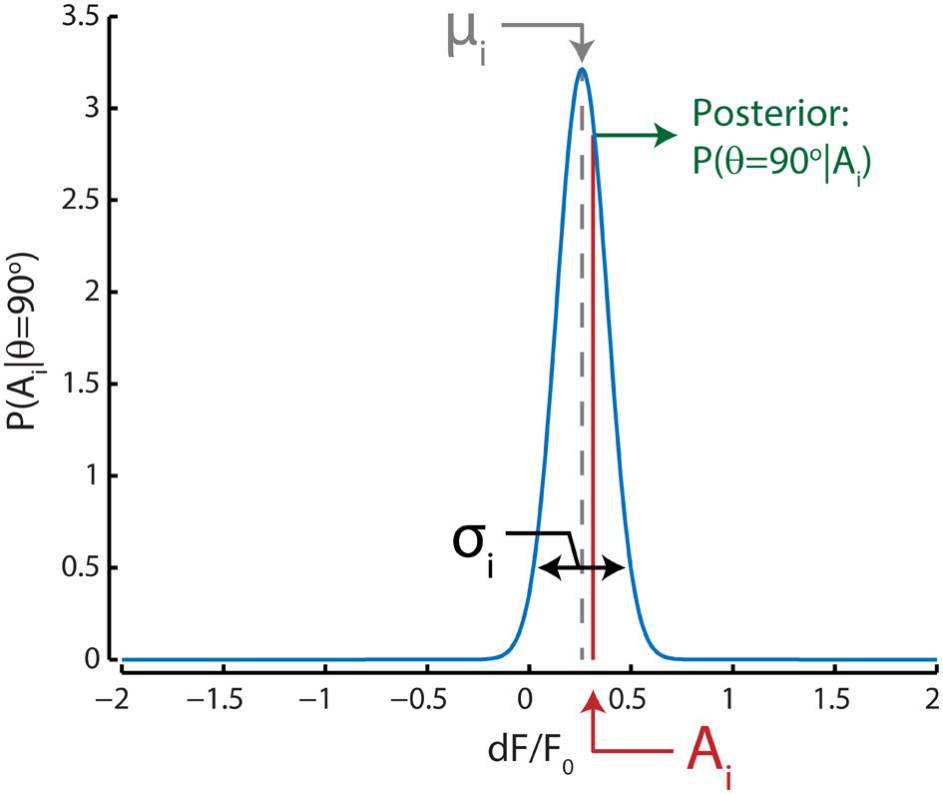
**Graphical representation of the Bayesian decoding scheme shows the process for one example neuron *i* for a single stimulus direction (90°) in one test trial**. The probability of responses to a 90° stimulus [P(A_i_ | θ = 90°)] for this neuron is approximated by a Gaussian distribution (blue curve) with a mean (μ_i_, gray) and standard deviation (σ_i_, black) equal to those estimated from training trials where this stimulus direction was presented. For this example to-be-decoded test trial, the neuron's activation level (A_i_, red) was close to the neuron's mean response to stimuli with a direction of 90 degrees; therefore the posterior probability [P(θ = 90° | A_i_), green] that the stimulus was 90 degrees is relatively high based on only this neuron's activation level and tuning. This procedure is repeated for all stimulus directions and the posterior probabilities for all neurons are multiplied. The stimulus direction with the highest probability is subsequently chosen as the maximum-likelihood (ML) read out.

The likelihood distribution for a certain direction, say θ = 90 degrees, for any neuron *i* was given by a Gaussian approximation based on the mean (μ) and standard deviation (σ) of its response to stimuli with that direction during training trials (Figure 2); in our case, we included all 80 trials to determine the likelihood distributions. Note that these Gaussians are not tuning curves, but rather an approximation of the *dF/F*_0_ response distribution for a single stimulus direction. The posterior probability for that direction can then be extracted by taking the value of the Gaussian likelihood distribution at the given neuronal activation level *A*. The population posterior probability distribution for that direction can be calculated by taking the product over the posterior probabilities for all *n* neurons:

(12) $$P\text{​}\left(\theta |A_{\text{pop}}\right)\propto \prod {}_{\text{i }=\;1}^{n}P{\left(\theta |A\right)}_{i}$$

Now the decoded direction can be read out by taking the direction with the highest probability.

### Jackknifing procedure

One question that can be addressed with these decoding algorithms is whether certain properties of a neuronal response correlate with decoding performance. We therefore performed a bootstrapping procedure (1000 iterations) per recording to select random groups of neurons (ranging in size from 2 to 15) from the whole datasets of 95–144 neurons. Each bootstrap resampling was followed by a jackknife procedure applied to all neurons in the randomly selected group to quantify the contribution of these single neurons to the decoding algorithm's performance. A jackknifing procedure provides insight into the contribution of a single neuron within a sample by taking away that neuron from the rest of the sample and comparing the properties (i.e., decoding performance) of the sample with and without this neuron. This procedure can be repeated for each neuron in the sample and yields a change in decoding performance per neuron that can be compared to other properties of the neuron. For example, jackknifing a certain neuron from a sample that has a high OSI might result in a different change in decoding performance than jackknifing a different neuron from the same sample having a low OSI. This way, the impact of several neuronal properties on decoding performance can be assessed. We normalized the decoding improvement effected by the jackknifed neuron for the number of neurons in the sample by defining the decoding improvement index D_i_ as

(13) $$D_{i}=ND-\left(N-1\right)D^{\neg i}$$

where *N* is the sample size, *D* the decoding accuracy of the algorithm using the entire cluster and *D*^¬*i*^ is the accuracy using the entire cluster except the jackknifed neuron (*i*). The decoding improvement was then binned and averaged over all points per bin from all 6 data sets (see Figure 6).

### Tuning property interdependency

Three important factors that may influence how much information on stimulus direction a neuron contributes to a neuronal cluster are the neuron's preferred orientation (PO), its signal correlation (SC) and its noise correlation (NC) with other members of the cluster. To further quantify if and how these properties show interdependencies, and vary with intersomatic distance (ID) in the imaging plane, we performed a pairwise comparison between neurons for these properties. For all neuronal pairs in all sessions we computed the SC, NC, ID and angular difference in preferred orientation (dPO). We then pooled data points from all sessions and performed a regression analysis to test if there were significant correlations between ID-SC, ID-NC, ID-dPO, SC-NC, SC-dPO and NC-dPO. Two properties were judged to be significantly interdependent when the 99% confidence interval for the regression slope did not overlap with 0. To determine the significance of a difference in slopes between dependent properties for a given independent property (either ID or SC), we z-scored the values of the dependent variable and performed another set of linear regressions. Because of this normalization, we could now quantitatively compare the relative slopes between ID-SC, ID-NC and ID-dPO, and between SC-NC and SC-dPO by assessing whether the 99% confidence intervals of these regression slopes overlapped.

### Removing biases in tuning property interdependency

As will be further elaborated in the Results section, it is possible that the previously described analysis shows significant relationships between neuronal properties that depend on mean differences in those properties between data sets, when in fact these properties are not significantly related within data sets. To control for such possible biases due to across-recording differences, we pooled all neuronal pairs into data bins for ID (0 to 200 with steps of 40 microns), SC (−0.7 to +0.7 with steps of 0.2) and NC (−0.175 to +0.275 with steps of 0.05). These bins were chosen so that at least 90% of all neuronal pairs per data set would be included in the analysis, with the number of points per bin still sufficiently large for robust data analysis. We then computed the minimal number of pairs for each bin for each calcium imaging data set and used this number as the resample size for a bootstrapping procedure (256, 305, and 84 pairs/bin/recording for ID, SC, and NC respectively). For each bootstrapping iteration (*n* = 1000), all bins received this number of randomly selected data points (neuronal pairs) from each data set, so that no across-recording differences in mean neuronal property values could lead to biases in the pooled resampled data set. We then performed a linear regression on each randomly sampled set and computed the 99% confidence intervals of the regression slopes to determine whether there was a significant correlation between two neuronal properties.

## Results

### The classical population vector fails at decoding moving directions

**Figure 3:**
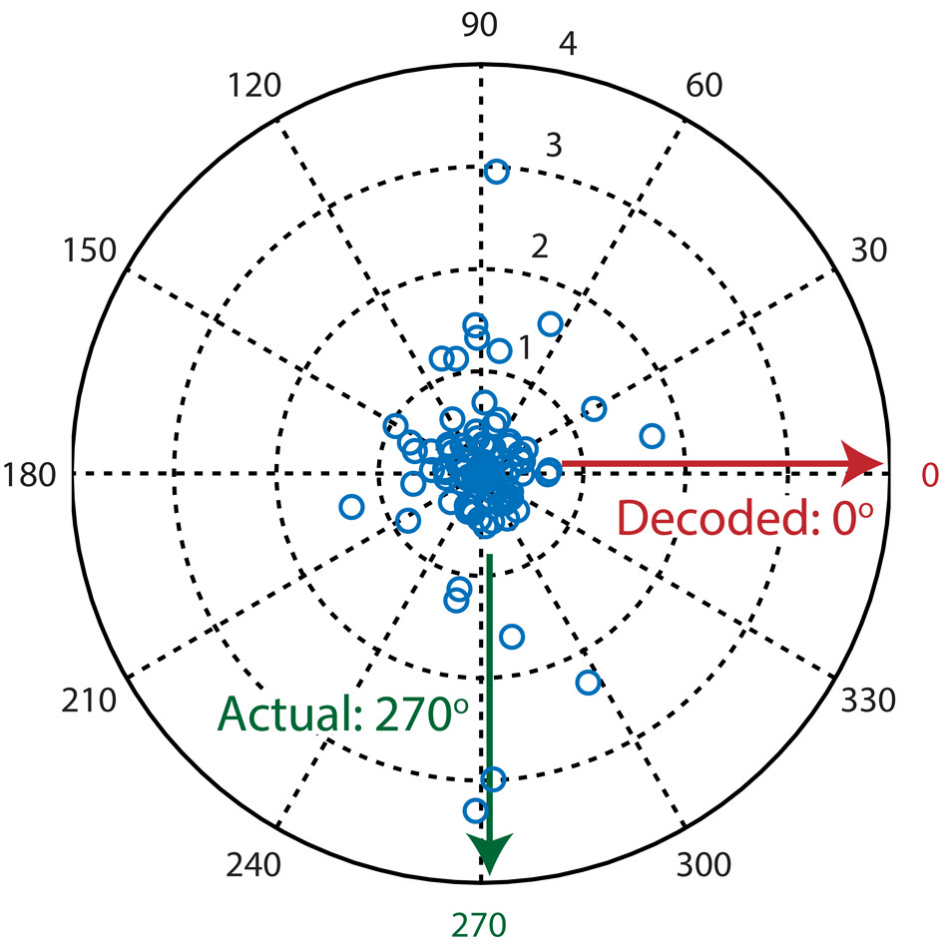
**Example decoding trial where the population vector algorithm fails to decode the presented stimulus direction**. Each blue circle in the polar plot is a single neuron where its preferred direction is represented by θ (angle) and its activation level (*dF/F*_0_) is represented by ρ (magnitude). The population vector algorithm's output is calculated by taking the circular mean over all vectors (neurons). Since most neurons are sensitive to two moving directions that are separated by 180°, calculation of the resultant vector leads to mutual cancellation of the two peaks at 90° and 270°, therefore resulting in an incorrectly decoded direction of 0°.

**Figure 4:**
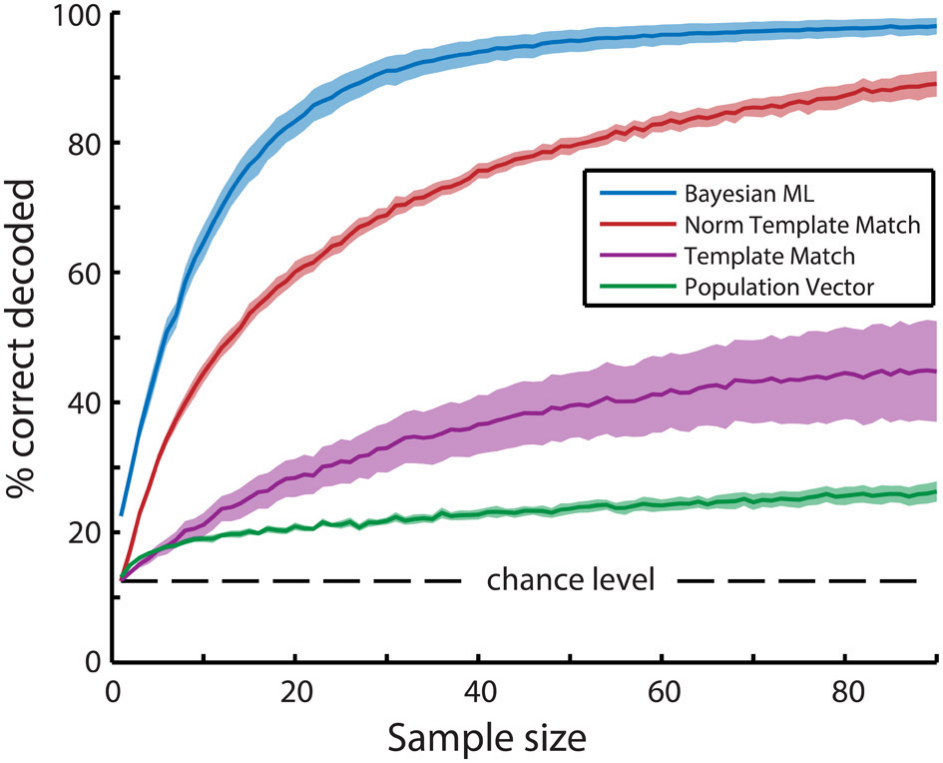
**Mean bootstrapped performance over all recordings (*N* = 6) reveals superior performance of the Bayesian maximum-likelihood (ML) direction decoding algorithm**. Bootstrapping was performed with 100 random resamplings with sizes ranging from 1 to 90 neurons. Dashed lines indicate standard error of the mean bootstrapped performance. A Three-Way ANOVA (algorithm, recording, sample size) revealed significant main effects of all three variables (*p* < 10^−20^). *Post-hoc t*-tests with Tukey-Kramer correction for multiple comparisons showed significant differences between all combinations of the 4 types of algorithms (*p* < 10^−10^). In descending order of performance, the tested algorithms are Bayesian ML (blue), Normalized Template Matching (red), Non-normalized Template Matching (purple), and Population Vector (green).

One of the simplest algorithms to decode neuronal population activity is the population vector method (see Methods; Georgopoulos et al., 1986). The only determinants of the contribution of a neuron's activity to the population vector are its preferred stimulus property (i.e., preferred direction) and activity level. Stimulus direction was decoded by calculating the resultant vector over all neurons (Figure 3). The performance of the population vector (Figure 4; green line), as measured by the percentage of trials that was decoded correctly, was above chance level, but this decoder clearly performed much worse than more complex algorithms. Moreover, the performance rose only slowly with the number of neurons in the sample, such that it would take a very large sample for its performance to catch up with the other algorithms.

### Template matching decoding reveals a strong sensitivity to highly active, highly variable neurons

Secondly, we tested the decoding performance of a template matching algorithm (Lehky and Sejnowski, 1990; Zhang et al., 1998; van Duuren et al., 2008, 2009). Its performance (Figure 4; purple line) was better than the population vector method, but it suffered from a significant bias, as neurons with higher response amplitudes dominated the algorithm. If the reliability of a neuron would scale linearly with its response amplitude to the stimulus (*dF/F*_0_), such a weighting might be beneficial, because the inherent bias incurred by high response amplitudes would favor reliable neurons. To examine whether there is indeed a bias and whether this bias poses a problem for the algorithm, a calculation of the Fano factor of the neuronal responses is in order (Fano, 1947).

The Fano factor is an index of variability and is defined as σ^2^/μ (the variance of the response divided by the mean). With neuronal spiking traces, simply pooling all spikes per neuron in time bins allows the calculation of a Fano factor per neuron from the mean spiking rates and variance in those bins. However, Fano factors calculated from the raw *dF/F*_0_ trace of calcium imaging data are biased. Although there is a linear relationship between *dF/F*_0_ and spiking activity for rates commonly observed in pyramidal cells (Kerr et al., 2005), the percentage increase in *dF/F*_0_ due to a single action potential can differ from one neuron to the next. This means that the Fano factor has a scale sensitivity that needs to be corrected per individual neuron. We therefore developed a non-scale-sensitive ratio Fano factor (r_Fano_) instead (see Methods).

**Figure 5:**
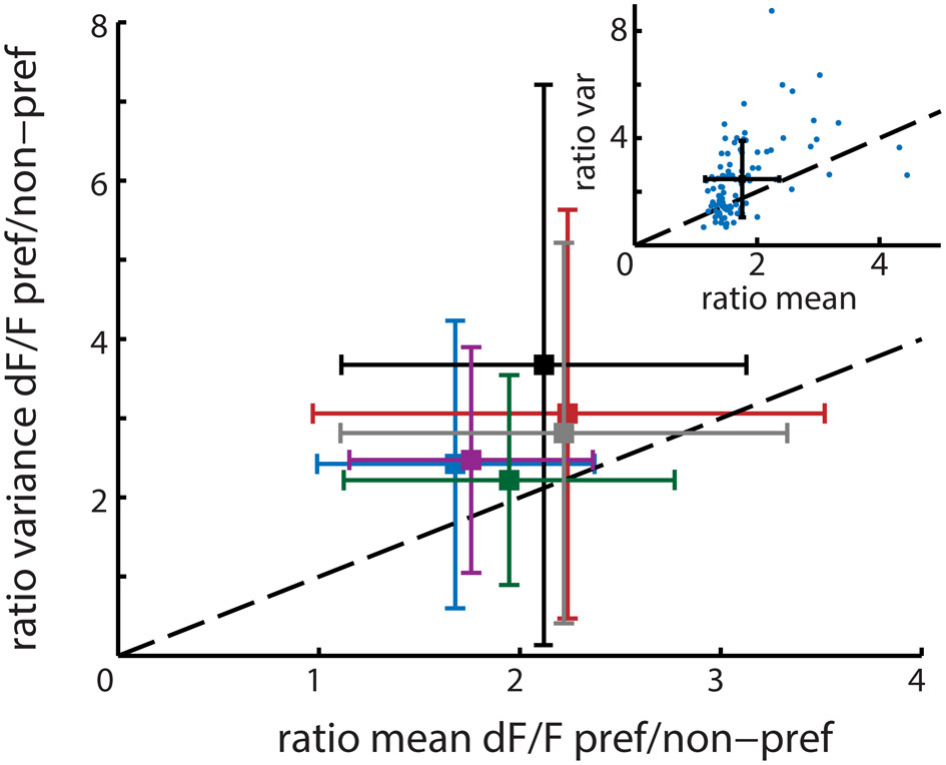
**Main figure shows the mean and standard deviation per recording session (*n* = 6 animals) in ratio variance (y-axis) and ratio mean (x-axis) activation level differences between time bins corresponding to the presentation of the neuron's preferred stimuli and non-preferred stimuli**. The mean population ratio-Fano (r_Fano_) is above the dashed diagonal and therefore higher than 1, indicating that on average the variance of a neuron increases more than its mean response when presented with its preferred stimulus. The six colors correspond to the different recordings. The upper right inset shows one example session where each single neuron is represented by a single point. The black cross shows the resultant mean ± *SD* for that session.

The mean r_Fano_ calculated over all 6 recordings was 1.39 with a standard deviation of 0.20 (see Figure 5), which is close to the Fano Factor of 1 for a theoretical Poisson process and in the range of the typically observed Fano Factors for experimental spiking data (Dayan and Abbott, 2001). This value is higher than 1, which implies that neurons with higher activation levels actually have lower reliability on average. This would suggest that the template matching algorithm is indeed prone to overrepresentation of highly active and relatively variable neurons.

The dominance of the template matching algorithm by neurons with high activity levels can be accommodated by taking the mean z-score normalized activation level across trials for all neurons instead of their actual activity level (see Methods). Consider the hypothetical case that two neurons are used for decoding, where one neuron is highly active and has a high Fano factor, while the other is less active, but has a lower Fano factor. The former neuron's mean stimulus-driven activation level over the entire recording is 1.0 *dF/F*_0_ with a standard deviation of 1.0 *dF/F*_0_ and its response to a certain stimulus is 2.0 *dF/F*_0_ on average, while the latter neuron's mean activation level is 0.5 *dF/F*_0_ with a standard deviation of 0.25 and this neuron's response to the same stimulus is 1.5 *dF/F*_0_ on average. In this case, both neurons' specific stimulus-responses are 1.0 *dF/F*_0_ higher than their average response, so the original template matching algorithm will weigh the activation levels of these neurons equally when determining the probability of this stimulus direction. However, the former, variable neuron's response to the stimulus would on average be one standard deviation away from its mean, while the latter, reliable neuron's response to the stimulus is four standard deviations away from the mean. The more reliable neuron therefore provides much more information about stimulus identity than the former neuron. Using z-scored activation levels therefore allows the template matching algorithm to take into account the reliability of the neurons involved. The performance of the normalized template matching algorithm (Figure 4; red line) is indeed clearly superior to the non-normalized template matching algorithm.

### Bayesian maximum-likelihood (ML) decoding with intermediate to large sample sizes shows near-perfect direction decoding

For further analysis of tuning property dependencies we created a Bayesian maximum-likelihood (ML) decoder. In contrast to the population vector and template matching algorithms we previously described, the ML decoder calculates posterior stimulus probabilities on a single neuron basis and then takes the product over probabilities contributed by all neurons per stimulus. Because the probabilities are computed on a single-neuron level and thereafter aggregated into population posterior probabilities, the ML decoder inherently corrects for differences in response reliability between neurons. The Bayesian ML decoder indeed easily outperforms all other decoding algorithms (Figure 4; blue line). We therefore performed all following decoding analyses with the ML decoder.

### Single neuron contributions to decoding accuracy depend critically on orientation selectivity index, signal correlations and noise correlations at larger neuronal sample sizes

**Figure 6:**
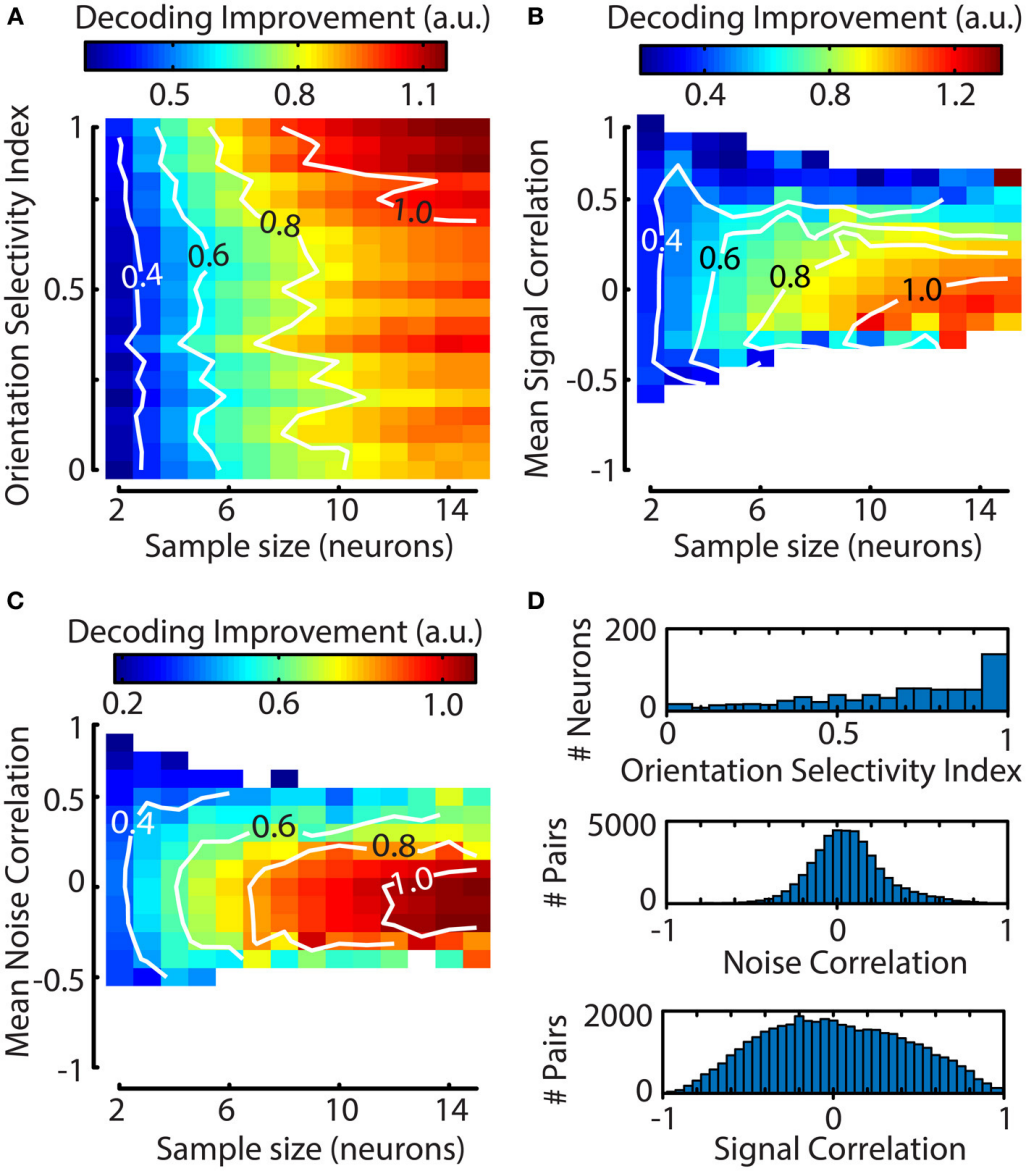
**The contribution of a single neuron to the decoding performance of a Bayesian ML algorithm depends on the neuron's orientation selectivity index (OSI) and its mean correlation with other neurons**. **(A–C)** Graphs were created by using a bootstrapping procedure for random cluster selection and a subsequent jackknifing procedure to quantify the impact of a single neuron's addition to the cluster. Contiguous contour lines are plotted in white for increased readability. **(A)** The influence of a neuron's OSI on the decoding performance of its cluster is stronger for larger clusters. Neurons with a higher OSI improve the algorithm's performance more. **(B)** The addition of a neuron that has a negative mean signal correlation (ρ≈−0.2 −0.1 for *N* = 10–15) with the rest of its cluster leads to a larger increase in decoding performance. **(C)** Slightly negative to low noise correlations (ρ≈−0.3 0 for *N* = 10–15) improve decoding performance; as with **(A,B)** this effect is stronger for larger sample sizes. **(D)** Population distributions pooled over sessions. Top panel; population distribution of orientation selectivity index (OSI), Middle panel; noise correlation, Bottom panel; signal correlation. The means for the distributions of noise and signal correlations do not correspond to the values that lead to maximum decoding improvement.

One question that can be addressed with a decoding algorithm is whether certain properties of a neuronal response have any effect on decoding performance. For instance, an intuitive hypothesis holds that decoding performance depends on a neuron's orientation selectivity index (OSI; see Methods). To investigate this further, a bootstrapping procedure with 1000 iterations was performed to select random clusters of neurons (2–15) and was followed by a jackknife procedure on all neurons per cluster to quantify the contribution of single neurons to the decoder's performance. We calculated each neuron's contribution to the decoding accuracy as an index normalized for sample size, allowing us to compare the effects over different sample sizes [see Equation (13); Figure 6A]. The results show that at small sample sizes a neuron's OSI has little effect on the decoding performance. However, at larger sample sizes, the addition of a neuron with a high OSI results in a larger improvement of the decoder's accuracy.

However, the OSI captures only a small part of the way a neuron is tuned to properties of visual stimuli. To more fully capture the similarity in visual responses between neurons we also calculated the mean pairwise signal and noise correlations between the jackknifed neuron and the other members of the sample. The results of this analysis show a clear dependence of decoding performance on the jackknifed neuron's mean signal correlation with the sample, where negative values are associated with higher decoding performance (Figure 6B). Similarly to the pairwise signal correlations, strong positive noise correlations had a deleterious effect on decoding performance (Figure 6C). For both the signal and noise correlations, the effect was stronger for larger clusters, indicating that response heterogeneity is important for efficient coding by large assemblies.

### Analysis of interdependence between response properties reveals a dissociation between stimulus tuning properties and random fluctuations

**Figure 7:**
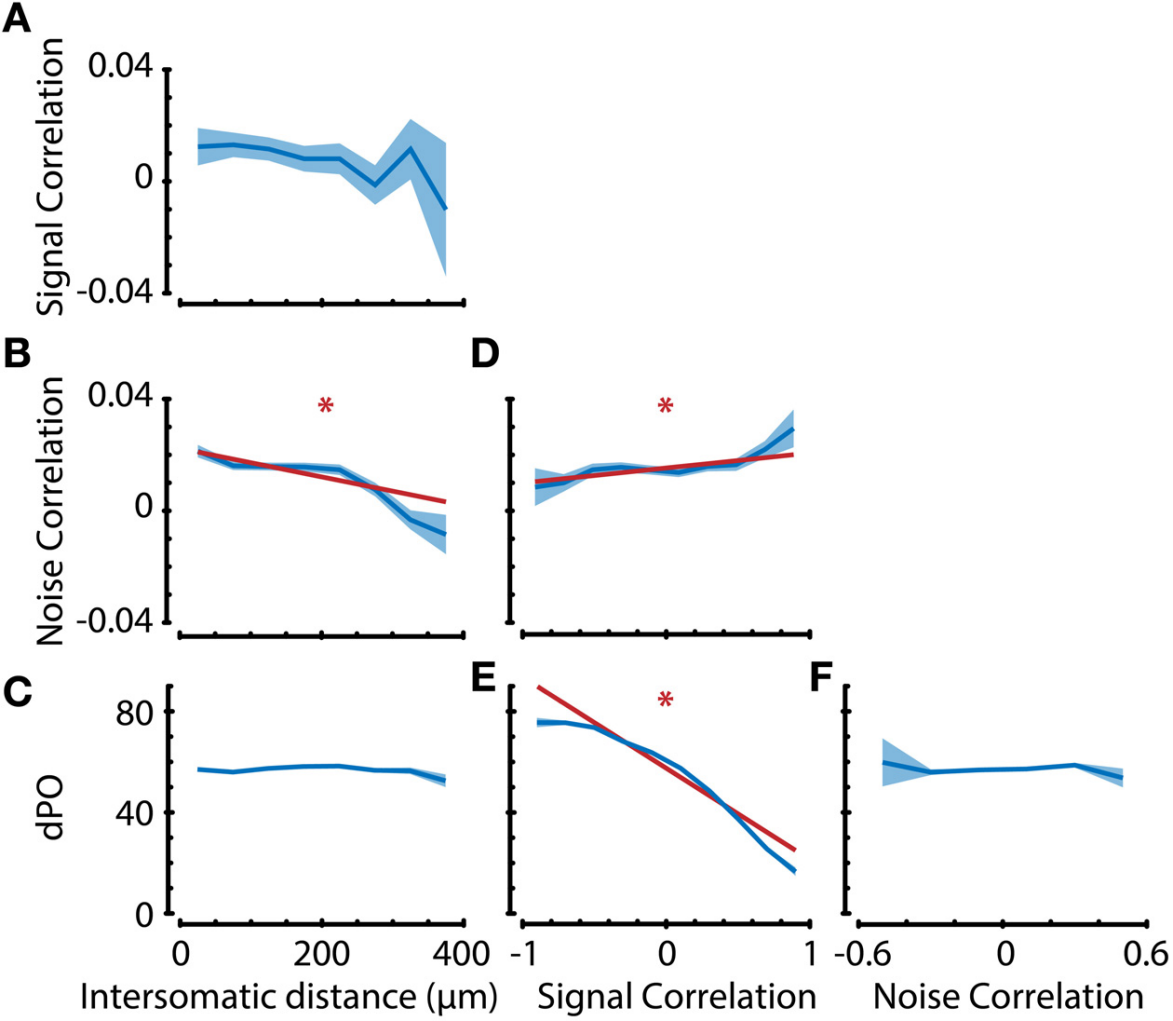
**Analysis of interdependence between response properties suggests dissociation in anatomical substrates of noise correlations and preferred orientation**. **(A–F)** Neuronal property interdependence analyses showing means per bin (dark blue line) with standard error (blue shaded area). Linear regression analyses were performed on the raw, unbinned data to check for significant correlations between properties. If the linear regression analysis indicated that the slope was significantly different from 0 with an α (significance level) of 0.01, the regression line (dark red line) was also plotted. **(A–C)** Signal correlation, noise correlation, and difference in preferred orientation (dPO) as a function of intersomatic distance (ID). Noise correlation, but not signal correlation or dPO, shows distance dependence. Subsequent comparison of the confidence intervals of regression slopes revealed a significant difference in ID-dependent slopes between NC-dPO (*p* < 0.01), but not SC-NC or SC-dPO. This suggests a different anatomical substrate of noise correlations and preferred stimulus direction. **(D)** Noise correlation is positively correlated with signal correlation (which entails multiple tuning properties), indicating that there is not a complete dissociation for all tuning properties. **(E)** dPO is negatively correlated with signal correlation and has a slope significantly different from NC **(D)** (*p* < 0.01), but **(F)** shows no dependence on noise correlation, providing further evidence for a dissociation of similarity in preferred orientation from pairwise noise correlation. ^*^p < 0.01.

The previous paragraph described the dependence of decoding accuracy on similarity in stimulus tuning properties (signal correlations and OSI) and random fluctuations (noise correlations). While these neuronal response properties may exert independent effects, it is also possible that they are strongly interdependent and might in reality have a single origin that is partly reflected in all response similarity indices. Because the spatial distance between cortical somata strongly influences the probability that two neurons are interconnected (Gilbert and Wiesel, 1983; Nicoll and Blakemore, 1993), we first investigated whether neuronal response properties depend on intersomatic distance. We therefore analyzed whether the angular difference in preferred orientation (dPO), signal correlation (SC) or noise correlation (NC) depend on the intersomatic distance (ID) by pooling all neuronal pairs from all recordings and performing a linear regression to test for significant interdependence (Figure 7).

As reported before in other animal species, such as cat (Ch'ng and Reid, 2010) and macaque (Smith and Kohn, 2008), we found that noise correlations significantly vary with intersomatic distance (Figure 7B; regression slope ≠ 0, *p* < 0.01), where pairs of neurons that are closer together show higher noise correlations. Noise correlations are assumed to reflect common input between neurons (Nienborg and Cumming, 2010), so one could hypothesize that if noise correlations are high, there should also be high similarities in the tuning properties of neurons. However, the spatial dependencies of preferred orientation difference and signal correlations were non-significant (Figures 7A,C), indicating that there might be a dissociation in origin between stimulus tuning properties and random fluctuations. It is important to note here that mice do not possess orientation tuning columns and that the spatial scale investigated is <400 microns, which is much smaller than for example the scale on which Smith and Kohn (2008) found a spatial dependency of correlations in macaque V1.

We next investigated the dependence of dPO on SC and NC to see if the similarity in stimulus tuning properties is correlated with the strength of random fluctuations (Figures 7E,F). As expected, we found that dPO is dependent on signal correlations (Figure 7E; regression slope ≠ 0, *p* < 0.01). However, dPO did not show any dependence on noise correlations (Figure 7F), which—in line with the distance dependence analysis—supports the conclusion that input fluctuations are dissociated from the inputs that determine a neuron's preferred orientation. The analysis of NC/SC dependence (Figure 7D regression slope ≠ 0, *p* < 0.01) verified previously published observations that NCs are positively correlated with SCs (e.g., Lee et al., 1998; Kohn and Smith, 2005; Cohen and Maunsell, 2009), suggesting that this dissociation is not complete.

To check whether the suggested dissociation still holds under more stringent statistical analysis, we tested whether the confidence intervals for the regression slopes were significantly different. After z-scoring the dPO, NC, and SC values in order to quantitatively compare the slopes for these properties, we found that the ID-dependent slopes of NC-dPO (*p* < 0.01), but not of SC-NC or SC-dPO were significantly different. Following the same approach, we also compared the signal correlation-dependent slopes of dPO and NC, and found them to be significantly different (*p* < 0.01). Taken together, these results indicate that a neuron's preferred orientation is dissociated from the similarity in random fluctuations in spiking activity. It also indicates that there are stimulus tuning aspects (such as bandwidth, peak-trough ratio, etc.) captured by signal correlations that are different from the neuron's preferred orientation, and that these aspects might have anatomical substrates similar to those of noise correlations (Figure 7).

When comparing property-dependent differences across sessions such as described in the previous paragraphs, a corrective measure must be employed to ensure that these significant inter-property dependencies cannot be explained by across-session differences. Suppose that we compare two hypothetical recordings where the mean noise correlations are higher for recording 1 than recording 2, and the field of view also differs between recordings. In this case, recording 1 has a field-of-view resulting in a mean intersomatic distance (ID) of 100 with a maximum ID of 200 microns and the other recording has a mean ID of 150 with a maximum ID of 300 microns. Even if there were no interdependence between noise correlation and intersomatic distance, a non-debiased analysis could detect a correlation, since all neuronal pairs with IDs more than 200 microns will come from session 2, which has a lower mean noise correlation. A regression analysis on the pooled neuronal pairs could then show a significant negative correlation where there is none. One way to correct for across-recording differences in mean or variance is to z-score the values per session and then pool the z-scored data sets. However, such a z-scoring approach does not correct for all biases. Using the same hypothetical example recordings as described above, z-scoring the intersomatic distance (ID) will *create* a bias rather than correct for it, because it would equate neurons with an ID of 100 in recording 1 with an ID of 150 in recording 2, since they are both equal to the recording-mean. To control for possible biases due to across-recording differences, we performed a debiased property interdependence analysis that takes a fixed and equal number of neuronal pairs from all recordings for several data bins (see Methods). The debiased analysis confirmed the results we obtained from the non-debiased regression analyses; ID-NC, SC-NC, SC-dPO showed significant linear correlations (*p* < 0.01), while ID-SC, ID-dPO, and NC-dPO did not. The neuronal property interdependencies we found are therefore not due to across-recording differences.

## Discussion

### Explaining the weak performance of the population vector method

The population vector exhibits the weakest performance of the tested algorithms, because it relies on two important assumptions that are violated. First, the population vector method assumes a uniform distribution of preferred directions over the encoded dimension. Clearly, decoding a stimulus direction with a sample size of one neuron will not yield a uniform distribution of preferred directions. This results in a decoded direction that is biased to the direction that is overrepresented by random resampling from the entire population. Moreover, neurons' preferred directions are not uniformly distributed in the visual cortex, but have a bias toward overrepresentation of cardinal directions (Kreile et al., 2011).

The second violated assumption is that each neuron has a single preferred direction. In V1 most neurons are selective for an axis of orientation (e.g., responding to both upward moving and downward moving) rather than one specific direction (e.g., responding to only upward moving), resulting in a tuning curve with two peaks rather than one (see Figure 3 for an example trial). When the stimulus-driven population activity is divided in two peaks that are separated by 180 degrees, taking a circular mean over the whole population will reduce the size of the signal that is extracted. This reduction takes place because two vectors with opposite directions will result in an average vector that has a magnitude equal to the magnitude in the preferred direction minus the magnitude in the opposite direction. When the population responses in the preferred and opposite direction have similar strengths, the resultant vector has a small magnitude and the decoded direction is especially prone to random imbalances in activation (noise). In essence, the bimodal nature of orientation tuning curves leads to a large decrease in signal-to-noise ratio (SNR) when decoding directions with a population vector. Therefore, although population vector decoding of orientations (Vogels, 1990) and of directions with only direction-selective and no orientation-selective neurons work well (Seung and Sompolinsky, 1993), direction decoding based on all (mixed) neurons shows very low accuracy. Since fixing or avoiding these two violations would require significant alteration of the population vector algorithm, we conclude that the classical population vector is not suitable for decoding visual stimulus directions using populations of V1 neurons.

### The critical dependency on input reliability suggests a neural mechanism for variability normalization

The template matching (TM) algorithm—which does not assume any particular shape of tuning curves—performed much better than the population vector method. However, our results showed that the TM decoder is highly sensitive to dominance by unreliable, highly active neurons. It has been previously reported that neuronal firing rate variability increases more than the mean spiking rate; i.e., neuronal Fano factors are usually >1.0 (Tolhurst et al., 1981; Baddeley et al., 1997; Dayan and Abbott, 2001). It has been shown that such codes could serve to maximize the spikecount entropy, and thereby the amount of information coded by neurons (Baddeley et al., 1997). However, this interpretation becomes problematic considering that the neuronal recordings in the previously mentioned studies were performed using extracellular microelectrodes, which are notoriously biased toward detecting only the most highly active neurons (Olshausen and Field, 1997; Wohrer et al., 2013). Because Fano factors >1.0 have a disproportionally large effect on highly active neurons, the increase in informational entropy might come at the cost of increased decoding complexity due to the discrepancy in variability over different neurons.

This would entail that the previously mentioned analyses of interactions between response variability and informational entropy (Tolhurst et al., 1981; Baddeley et al., 1997; Dayan and Abbott, 2001) hold for only a small subset of the entire population of neurons. The results presented in the current paper are based on two-photon calcium imaging; a non-biased recording technique. Our analyses confirm the previous observations that neuronal Fano factors are >1.0 for highly-active, but also for less active neurons. However, our current analyses also show that normalizing neuronal data by Z-scoring improves the decoder's accuracy tremendously. While this operation does not negate response non-stationarity, it does account for differences in spiking rates and Fano factors. This stimulus-invariant response normalization is somewhat reminiscent of the more general principle of population divisive normalization that has already been used to explain a host of other neuronal response properties, such as contrast-invariant neuronal responsiveness, attentional modulation, stimulus adaptation and multi-areal time-dependent attentional progression effects (Lee and Maunsell, 2009; Reynolds and Heeger, 2009; Ringach, 2010; Montijn et al., 2012; Benucci et al., 2013). Possibly, the stimulus-invariant variability normalization we applied to enhance the template matching algorithm's performance represents a neural mechanism enabling divisive normalization at a neuronal population scale.

One way variability normalization might be neurally implemented is through a simple Hebbian plasticity rule, if neurons with a high variance in firing rates also produce unreliable spike timings. The hypothesis holds that such neurons are presynaptic to neurons in higher visual areas and that their input strength inversely correlates with their firing-rate variability. Given the observation that synaptic plasticity is strongly dependent on the exact timing of spikes, as in spike-timing dependent plasticity (STDP) (Bi and Poo, 1998), this interpretation makes two testable predictions: (1) neurons with high variances in stimulus-driven spike rates have high variances in spike timing; and (2) the synaptic strength between orientation-tuned neurons and their targets depends on the spike rate reliability over trials of the presynaptic neurons.

### Bayesian maximum-likelihood decoder shows robust performance

The Bayesian maximum-likelihood (ML) decoder—with its assumption of Gaussian response distributions within stimulus types (Figure 2)—performs even better than the template matching algorithm, even though Gaussian distributions are often poor approximations of neuronal response distributions. It would seem that in our case the exact shape of the response distribution of any neuron is less important than the reliability of its response. The Bayesian ML decoder also shows robust performance despite another violated assumption. In our case, the ML decoder assumes statistical independence between neurons, because of its product-rule transformation from individual posterior probabilities per neuron to a population posterior probability. Even though we have shown that inter-neuronal noise correlations are non-zero, the decoder algorithm shows an accuracy higher than that of all other tested algorithms, and reaches >90% correct with a fairly small sample size (>28 neurons).

### In larger populations narrowly tuned neurons encode more information than broadly tuned neurons

Results from jackknifing single neurons from a larger cluster show that orientation selectivity (OSI), signal correlations and noise correlations can be important factors influencing decoding performance. Orientation selectivity has a strong influence on a single neuron's information contribution in large clusters, but has hardly any relation to the contribution in small clusters. This observation can be related to the idea that sharply tuned neurons would contribute more information than broadly tuned neurons if the population size is sufficiently large to offer a complete coverage of the stimulus dimension (Hinton et al., 1986; Seung and Sompolinsky, 1993; Schoups et al., 2001; Kang et al., 2004; Montemurro and Panzeri, 2006). Within this framework, the relative unimportance of OSI at small sample sizes can be explained by the trade-off between orientation selectivity and having a tuning curve that encompasses a high proportion of the entire stimulus dimension ([0–360] degrees). In the extreme case of a sample size of 1 neuron, a highly selective neuron that responds to only 1 stimulus direction out of 8 will provide much information about 1/8th of all stimuli, but leaves the decoder to randomly guess in all other cases, while a more broadly tuned neuron would allow the decoder to make educated guesses in all 8 cases. With larger sample sizes and highly selective neurons, the chance decreases of having gaps in the representation of stimulus space, while these cells would still offer the benefits of high selectivity.

The idea that narrowing a neuron's tuning curve often increases its encoded information has been challenged more recently, but no definitive conclusions have yet been reached. For one, a computational study comparing two models for orientation selectivity that either included or excluded cortical sharpening of tuning curves reported that the amount of information coded by neurons is strongly dependent on the amount and structure of noise covariance (Seriès et al., 2004). While its results were striking, an important confound was that the output of the model was constrained to produce identical neuronal tuning curves. In essence, the study looked at the effect of cortical tuning curve sharpening, given the same output tuning curve of cortical neurons before and after sharpening, by changing the input tuning curves (LGN afferents). However, a more appropriate comparison would have been the comparison of coded information between pre- and post-sharpened tuning curves of cortical neurons, given the same LGN afferent tuning curves.

Another consideration regarding the optimal shape of tuning curves is that the stimulus information relies solely on the Fourier amplitude spectrum of the orientation tuning curve and not necessarily on other tuning curve properties that give a lower-order approximation for this Fourier-space tuning curve, such as bandwidth (Ringach, 2010). This would suggest that the narrow-vs.-wide debate, including some seemingly contradictory results regarding changes in bandwidth (e.g., Yang and Maunsell, 2004; Goltstein et al., 2013), might simply result from looking at parameters that describe tuning curves in a way that does not correctly capture the properties that are most important to their information coding potential. Fully capturing a neuron's information coding potential in a single—or even a handful of—tuning curve parameters might very well be impossible.

Perhaps even more importantly, the shape of neuron's tuning curve that allows maximum information coding potential is heavily dependent on the distribution of tuning curves within its population, and tuning curves within sensory cortical populations are very heterogeneous (Wohrer et al., 2013). Computational studies that look into how tuning curve properties influence the maximum amount of encoded information are important to further our understanding of stimulus coding in the visual system, but their often relatively homogeneous population of neuronal tuning curves and sometimes arbitrary parameter sets mean that one should be cautious in applying their theoretical findings too readily as a realistic model of sensory processing.

An important caveat to our observation that high OSI leads to higher decoding accuracy in large samples is that the less orientation-selective neurons we measured could have a lower signal-to-noise ratio (SNR), which would thereby convey a low orientation selectivity, as well as the measured, but not necessarily neurally coded information. To draw definitive conclusions, one would have to correct for neuronal SNR while analyzing the dependence of decoding accuracy on orientation selectivity, which could be an interesting subject for future validative research. Despite this caveat, our results are consistent with the more classical assumption that at least under our experimental conditions, greater orientation selectivity increases the amount of encoded information for larger populations of neurons.

### Uncorrelated, heterogeneous neuronal responses increase the amount of encoded information

Our results also show that the effect of signal- and noise correlations on population coding is sample-size dependent. Moreover, neurons that are added to a population increase the population information coding most when their response is dissimilar from the extant population (negative signal correlations) and they are uncorrelated (noise correlations near zero). Heterogeneity of a population becomes more important as the neurons' representation of stimulus space gets crowded, because there are more neurons to represent the same space. This also means that for decoders that do not take into account the entire covariance structure of their input population, noise correlations are detrimental. To further validate if the negative effect of noise correlations on decoding we present in this paper is truly detrimental or could be remedied by taking into account the neuronal correlation structure, it would be an option to create a decoder algorithm that takes into account this covariance matrix. However, accurately estimating the covariance matrix of a group of 90 neurons that respond to 8 different stimulus types would require the fitting of eight 90-dimensional multivariate Gaussians. Since the experiments we performed have only 10 repetitions per stimulus type, this would most probably result in poor fits of the covariance matrix. If neurons can indeed take into account the entire covariance matrix of their synaptic inputs (which can consist of up to 5· 10^7^ covariance values Huttenlocher, 1979), this would mean that although our results are strongly indicative of detrimental effects of noise correlations on population coding in V1, they do not offer conclusive evidence.

While extracting a proper covariance matrix from experimental data on populations of neurons is extremely difficult, several modeling studies have addressed the effects of correlation on population coding. One approach would be to create a neuronal network model and fit the model's parameters to experimentally measured data. For example, Pillow et al. (2008) showed that a computational model can decode 20% more information from experimentally measured retinal ganglion cell activity when it does not assume independence of the cells' responses. In general, when the signal correlation and noise correlation of a neuronal pair have opposite signs (i.e., negative vs. positive correlations); encoding fidelity can be improved (Averbeck et al., 2006). However, since high signal correlations between pairs of neurons tend to often also show high noise correlations (Lee et al., 1998), the theoretical possibility of increased performance from correlations might in practice not apply to neural systems in the cortex.

To settle this debate more definitively, two matters will have to be addressed. First, future research would have to determine whether neuronal pairwise noise correlations in V1 are stationary up to a point where synaptic plasticity could keep up with changes in the covariance matrix. If noise correlations are indeed adequately stable over time, a sufficiently large number of functionally connected neurons would then have to be measured over a relatively long time period, so that enough neurophysiological measurements can be collected to accurately estimate the covariance matrix of that group of neurons. The actual action potential firing of these neurons would then have to be compared to the theoretically predicted pattern when the neurons are decorrelating their synaptic inputs. Suffice it to say, this is unfortunately not possible with the currently used data set, but hopefully technical advances in ensemble recordings will make these kinds of measurement possible in the near future.

### Noise correlations and stimulus tuning properties may have different anatomical substrates

Noise correlations, but not signal correlations and angular preferred orientation difference, depend on intersomatic distance (Figure 7). This could indicate that noise correlations reflect common input (Nienborg and Cumming, 2010), in the sense that they are indicative of the temporal fluctuations in global synaptic barrages on their many dendritic arbors, but that tuning properties arise from mechanisms unrelated to overall fluctuations in spiking activity. In other words, robust visual response properties may depend on relatively reliable connections, while noise correlations are more related to non-specific global barrages conveyed by locally correlated inputs that become uncorrelated across anatomical space.

This last interpretation is in line with other research showing that noise correlations can be strongly influenced by more general modulatory processes, such as anesthesia, attention and arousal (Greenberg et al., 2008; Mitchell et al., 2009; Pennartz et al., 2013). It is also supported by the observation that layer 2/3 and layer 5 neurons display relatively high spiking correlations (mean around 0.1), while populations within L4 show correlations around 0.01; almost an order of magnitude smaller (Smith et al., 2012). As has been suggested before, horizontal cortico-cortical connections may be necessary for noise correlations (Ts'o et al., 1986; Smith et al., 2012). Such recurrent circuitry is present in superficial and deep layers, but probably not in layer 4 (Gilbert and Wiesel, 1983; Douglas and Martin, 2004), which may explain the difference in magnitude of noise correlations and provides further evidence that noise correlations might indeed have specific anatomical substrates. Moreover, it has been reported that L2/3 orientation tuned neurons in mouse V1 receive relatively more inputs from neurons that are themselves tuned to that orientation (Ko et al., 2011), which makes it plausible that stimulus tuning depends on specific synaptic connections.

The idea that stimulus tuning and noise correlations are based on different anatomical configurations also explains why we found a dissociation between the spatial dependence of noise correlations on the one hand, and the non-spatial dependence of dPO and signal correlations on the other. A testable prediction that follows from our hypothesis is that synaptic connections between similarly-tuned neurons should be stronger or more reliable than connections between differently-tuned neurons; meaning that the EPSPs they elicit have higher amplitudes or should occur in closer temporal proximity to the EPSPs of other similarly-tuned synapses connected to the same neuron, which would lead to more efficacious temporal summation. Because those synapses might also be originating from layer 4 neurons, rigorously testing the hypothesis would require simultaneous patching of layer 4 and layer 2/3 neurons *in vivo*.

To conclude, we have provided in-depth descriptions of several analysis methods that can be used to investigate population coding, including several of their drawbacks and advantages, and we have suggested how to avoid problems and biases conveyed by some of these methods, as applied to V1 data. The analyses reveal the importance of input reliability for reading population codes, suggest a role for response heterogeneity and decorrelation in population coding, and show there is a difference in anatomical intersomatic distance dependence between pairwise noise correlations and differences in preferred orientation. We have interpreted these results within a neurophysiological framework and presented testable predictions that follow from the hypotheses that Hebbian plasticity might lead to spiking variability normalization, and that noise correlations and properties underlying directional tuning arise from segregated anatomical substrates.

### Conflict of interest statement

The authors declare that the research was conducted in the absence of any commercial or financial relationships that could be construed as a potential conflict of interest.
